# Low Birth Weight Male Guinea Pig Offspring Display Increased Visceral Adiposity in Early Adulthood

**DOI:** 10.1371/journal.pone.0098433

**Published:** 2014-06-13

**Authors:** Ousseynou Sarr, Jennifer A. Thompson, Lin Zhao, Ting-Yim Lee, Timothy R. H. Regnault

**Affiliations:** 1 Department of Obstetrics and Gynecology, Children's Health Research Institute, Lawson Health Research Institute, Western University, London, Ontario, Canada; 2 Department of Physiology, Georgia Regents University, Augusta, Georgia, United States of America; 3 Departments of Medical Imaging, Medical Biophysics, and Oncology, Western University, London, Ontario, Canada; 4 Lawson Imaging, Lawson Health Research Institute, London, Ontario, Canada; 5 Robarts Research Institute, London, Ontario, Canada; 6 Department of Physiology and Pharmacology, Western University, London, Ontario, Canada; University of Warwick – Medical School, United Kingdom

## Abstract

Uteroplacental insufficiency (UPI)-induced intrauterine growth restriction (IUGR) predisposes individuals to adult visceral obesity. We postulated that low birth weight (LBW) offspring, from UPI-induced IUGR pregnancies, would display a visceral adipose lipogenic molecular signature involving altered gene expression, phosphorylation status of proteins of the lipid synthesis pathway and microRNA (miR) expression profile, occurring in association with increased visceral adiposity. Normal birth weight (NBW) and LBW (obtained by uterine artery ablation) male guinea pig pups were fed a control diet from weaning to 145 days and sacrificed. Despite being lighter at birth, LBW pups displayed body weights similar to NBW offspring at 145 days. At this age, which represents young adulthood, the relative weights of LBW epididymal white adipose tissue (EWAT) and lipid content were increased; which was consistent with adipocyte hypertrophy in the LBW offspring. Additionally, the mRNA expression of lipid synthesis-related genes including acetyl-CoA carboxylase 1 (ACC1), diglyceride acyltransferase 2 (DGAT2) and peroxisome proliferator-activated receptor gamma 1 (PPARγ1), was increased in LBW EWAT. Further, LBW EWAT displayed decreased phospho-ACC (Ser79) and phospho-PPARγ (Ser273) proteins. Moreover, the mRNA expression of hormone-sensitive lipase (HSL) and fatty acid binding protein 4 (FABP4), both involved in promoting adipose lipid storage, was increased in LBW EWAT. Finally, miR-24 and miR-103-2, miRs related to adipocyte development, were both increased in LBW EWAT. These findings indicate that, following an adverse *in utero* environment, lipid synthesis-related genes and miR expression, along with phosphorylation status of key regulators of lipid synthesis, appear to be chronically altered and occur in association with increased visceral adiposity in young adult IUGR male offspring.

## Introduction

Uteroplacental insufficiency (UPI), a complication of pregnancy due to a failure of the placenta to deliver adequate nutrients and oxygen to the fetus, is responsible for the majority of cases of intrauterine growth restriction (IUGR) in industrialized countries [1]. Human studies have implicated low birth weight consequent to IUGR as a risk factor for the development of later obesity [2]. IUGR adults have a relative increase in adipose tissue and tend to acquire more visceral white adipose tissue (VAT) but less subcutaneous adipose tissue (SAT) relative to total fat mass [3], [4]. Increased VAT mass is associated with insulin resistance and is a strong risk factor for the development of type 2 diabetes [5]. This constitutes a major component of metabolic syndrome, one of the leading public health challenges worldwide [6], [7].

Adipose tissue mass is determined by the interplay of fatty acid synthesis (lipogenesis), fatty acid uptake and β-oxidation processes and the breakdown of adipose triglycerides into glycerol and fatty acids (lipolysis) [8]. Adipose lipogenesis occurs either as *de novo* synthesis of fatty acids from non-lipid substrates such as glucose, acetate and lactate (also known as *de novo* lipogenesis or DNL), or as a consequence of re-esterification of free fatty acids with glycerol.

Adipose DNL is under the control of two key enzymes: acetyl-coenzyme A carboxylase (ACC) and fatty acid synthase (FAS) [9]. In humans and other mammals, ACC exists in two isoforms, ACC1 and ACC2. While ACC2 is highly expressed in oxidative tissues, such as the skeletal muscle and the heart, ACC1 is expressed mostly in lipogenic tissues including adipose tissue with the function of catalyzing the carboxylation of acetyl-CoA to produce malonyl-CoA. Malonyl-CoA is used as a building block to extend the chain length of fatty acids in two carbon increments, a process catalyzed by FAS [9]. ACC and FAS are regulated by the activation status of AMP-activated protein kinase (AMPK) where activation (phosphorylation at threonine 172 (Thr172) on the α-subunit) of AMPK results in inhibition of fatty acid synthesis via repressed expression of the ACC1 and FAS genes, combined with direct phosphorylation of ACC1 at serine 79 (Ser79) [10]. Further control of ACC and FAS expression and activity has been reported through the action of the transcription factor sterol regulatory element-binding protein 1 (SREBP1) and nuclear hormone receptors, including the nuclear peroxisome proliferator receptor γ (PPARγ) [11]–[13]. As with AMPK and ACC, phosphorylation status is now understood as being critical in determining activity and, in adipose tissue, decreased PPARγ phosphorylation at serine 273 (Ser273) creates a constitutively active PPARγ state in visceral adipose tissue [14]. While there is now a greater understanding of how the phosphorylation status of stress sensors and nuclear hormone receptors, such as AMPK and PPARγ, impacts adipose tissue mass dynamics, new upstream regulators such as non-coding microRNAs, have emerged in the literature. MicroRNAs (miRNAs or miRs) are implicated in the regulation of genes that are involved in adipogenesis (miR-24) [15], [16] and adipocyte enlargement (miR-103) [17]. Specifically, other miRs, miR-27a and miR-27b have been shown to play an important role in PPARγ expression [18]–[20], potentially highlighting these miRs as key regulators of PPARγ expression and ultimately impacting adipogenesis and/or DNL activity in adipose tissue.

In addition to DNL, the uptake and storage of circulating free fatty acids by adipocytes and re-esterification of fatty acids from intraadipocyte lipolysis also promote triglyceride synthesis and accumulation of adipose tissue [21], [22]. These processes involve a number of different proteins including the Cluster of Differentiation 36 (CD36) translocase, fatty acid binding protein 4 (FABP4), adipose triglyceride lipase (ATGL), hormone-sensitive lipase (HSL) and diacylglycerol acyltransferases DGAT1 and DGAT2 [23]–[27]. As with DNL, the regulation of these fatty acid metabolism proteins also involves nuclear receptors and miRs. Specifically CD36 has been proposed as direct target of PPARγ [13] and PPARγ plays a central role in regulating DGAT1, HSL and ATGL expression in white adipose tissue [28]–[30]. Additionally, FABP4 expression has been demonstrated to be under the control of miR-24 and miR-378 *in vitro* in preadipocytes and adipocytes respectively [31], [32].

These combined reports highlight the complex regulation of adipose tissue mass. The regulation of these proteins involved in fatty acid synthesis and metabolism has been well studied in adverse situations of excessive VAT accumulation, such as obesity [33]–[36]. To date, studies of potential programming impacts, as a result of an adverse *in utero* environment and the subsequent outcome on later life processes are only now emerging. The *in utero* environment is a critical regulator of many later life metabolic parameters. Indeed, with reference to adipose development, a decreased percentage of phosphorylated AMPK in omental adipose tissue in parallel with unchanged omental adipocyte size and omental fat mass have been reported in twenty-one-day-old IUGR lambs [37]. These data suggest that as a result of an *in utero* or early postnatal environment, phosphorylation changes persist into later life, setting the stage for later increased lipogenesis. Additionally, an increased accumulation of retroperitoneal, epididymal and perirenal white adipose tissues has been reported in association with the increased expression of lipogenic genes FAS and ACC, in adult male IUGR rats [38], [39], further suggesting a programmed increase in visceral adipose AMPK/ACC/FAS activation following an adverse *in utero* environment. Interestingly, these changes in the expression of lipogenic genes occurred in conjunction with increased PPARγ [39]. These studies highlight the concept that the *in utero* environment plays a critical role in programming later life adipogenesis and lipogenesis processes, through *in utero* induced programmed changes in, not only regulation of gene expression and its transcription, but also in conferring important post-translational modifications, many of which may have lifelong programmed implications.

Given that the *in utero* programming of later life metabolic function is critical to our understanding of later life disease evolution as well as the development of IUGR-associated obesity constituting a leading public health concern, further elucidation of the molecular origins of visceral obesity in IUGR/LBW adult offspring is warranted. Therefore we sought to investigate the interplay between several critical regulators of adipose lipid metabolism in LBW offspring from UPI-induced IUGR. We postulated that LBW young adult offspring would display an altered adipose molecular signature of genes, miRs and post-translational modifications of proteins of the lipid synthesis pathway, occurring in association with increased visceral adiposity.

## Materials and Methods

### Ethics Statement

Animal care, maintenance, and surgery were conducted in accordance with guidelines of the Canadian Council on Animal Care. The University of Western Ontario Animal Use Subcommittee approved all procedures.

### Animals

Time-mated pregnant Dunkin-Hartley guinea pigs (Charles River Laboratories, Wilmington, MA, USA) were housed in a temperature (20°C) and humidity (30%) controlled environment with a 12 h light–dark cycle and had access to guinea pig chow and tap water provided *ad libitum*.

All pregnant guinea pigs underwent uterine artery ablation (UAA) at mid-gestation (∼32 days, term ∼67 days) in order to induce UPI and IUGR as previously described [40]. Briefly, sows were induced in an anesthetic chamber (4–5% isoflurane with 2 L/min O_2_, followed by 2.5–3% isoflurane with 1 L/min O_2_ for maintenance). Immediately after induction, a subcutaneous injection of Robinul (Glycopyrrolate, 0.01 mg/kg, Sandoz Can Inc., Montreal QC) was administered. A midline incision was made below the umbilicus in order to expose the bicornate uterus and the number of fetuses was noted. At that time, arterial vessels were identified and every second branch was cauterized using an Aaron 2250 electrosurgical generator (Bovie Medical, Clearwater, FL). Immediately following surgery, a subcutaneous injection of Temgesic (Buprenorphine, 0.025 mg/kg, Schering-Plough Co., Kenilworth NJ) was administered. Sows delivered spontaneously, at which time, the offspring were weighed and pups weighing less than 85 grams in each litter were defined as low birth weight (LBW), whereas those classified as normal birth weight (NBW) weighed more than 90 grams. LBW and NBW pups remained with their dams during a 14-day lactation period. Five days prior to weaning, all the pups were introduced to the control diet (Harlan Laboratories TD.110240: 21.6% protein, 18.4% fat, 60% carbohydrates) and weaned on postnatal day 15 postnatal. At weaning and thereafter, male LBW (n = 5) and NBW (n = 7) offspring were given *ad libitum* access to the control diet and tap water, and housed in individual cages. Pups were weighed daily until postnatal day 50, after which weights were recorded twice weekly. Food intake was measured daily throughout the experiment. Only males were used for this study as changing estrogen levels in the estrous cycle acutely modulated adiposity and lipid metabolism in parametrial fat pad [41].

### Computed Tomography (CT) Measurements

At ∼120 days of age, *in vivo* computed tomography was undertaken to quantify total adipose tissue, total muscle and bone of the whole body (from the proximal tibia to the base of the skull) [42]. Anaesthesia in animals was induced in an anesthetic chamber (4–5% isoflurane with 2 L/min O_2_) and maintained with 1.5–3% isoflurane. Subsequently, images were taken at 1.25 mm slices, with voltage and current settings at 80 kV and 100 mA respectively, using a Discovery CT750 HD scanner (GE Healthcare, Mississauga, ON). Using an application developed in MATLAB (The Mathworks Inc., Natick, MA), adipose tissue, muscle and bone were differentiated by thresholding CT image slices of the guinea pig [43]. By using pixel size and thickness of each CT image slice, volumes (cm^3^) of adipose tissue, muscle and bone were calculated for each image slice and summed for each tissue type individually. The summed volume of each tissue type was normalized to body weight and expressed as a percentage of total body volume.

### Adipose Tissue and Blood Sampling

At postnatal day 145, which corresponds to young-adulthood [44], overnight fasted offspring were sacrificed by CO_2_ inhalation. The abdominal cavity was opened and blood samples from the inferior vena cava were immediately collected in EDTA (BioShop Canada Inc., Burlington, ON), subjected to 2000× g centrifugation for 15 min at 4°C and plasma stored at −80°C for later analysis. Epididymal white adipose tissue (EWAT) surrounding the epididymis, projecting anteriorly in the intra-abdominal cavity along the peritoneum, a major visceral adipose tissue in the guinea pig [45] was removed and weighed and a portion was frozen in liquid nitrogen for later molecular analysis. For histological analysis, a portion of EWAT was slow-frozen in cooled isopentane (Sigma-Aldrich Canada Ltd., Oakville ON). All samples were then stored at −80°C until analysis. Retroperitoneal and mesenteric adipose tissues were also weighed, snap frozen and stored at −80°C. As the relative weight of retroperitoneal and mesenteric depots was unchanged between NBW and LBW offspring (0.007±0.001 versus 0.009±0.003, p = 0.34 and 0.012±0.002 versus 0.014±0.003, p = 0.5 respectively), only the study of molecular signature of EWAT was undertaken.

### EWAT Lipid Content and Histological Determinations

Lipid content in frozen EWAT (250 mg) was determined using the chloroform/methanol extraction method [46] and expressed as relative to the wet tissue. For cell morphology, slow-frozen EWAT samples were fixed in 4% paraformaldehyde (Billerica, MA) for 12 hours overnight. The next day, fixed tissues were processed using a Leica ASP300 processor (Leica Microsystems, Nussloch) that included gradual ethanol incubation, xylene washes and paraffin infiltration through a 13 hour cycle. Tissues were then embedded in paraffin using a Shandon Histocentre 3 (ThermoFisher Scientific, Gormley, ON). Three serial cross-sections (5 µm-thick) per animal were cut on a Leica RM2255 microtome (Leica Biosystems, Nussloch). Hematoxylin and eosin (H&E)-stained cross-sections were captured on a microscope (Leica DMIL LED, Leica Microsystems CMS GmbH, Wetzlar) at 10× magnification using Leica Application Suite version 3.8.0 (Leica Microsystems, Heerbrugg). Individual areas of about 400 adipocytes in the three sections were measured using Image-Pro Plus software version 7.0 (Media Cybernetics Inc., Bethesda, MD). Results corresponding to the mean area (K) determined on the three sections of each adipose tissue sample were expressed as the mean diameter (d) in µm, considering adipocytes as spherical cells and following the formula 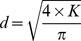 where, π = 3.142. The number of adipocytes per gram of tissue was calculated by dividing the total lipid content per gram of tissue (described above) by the mean estimated volume (V) of the adipocytes calculated as follow V (µm^3^) = 4/3×π×R^3^ where R = d/2.

### RNA Extraction

For total RNA extractions (mRNAs and microRNAs), EWAT samples (∼200 mg each) were homogenized in 1 ml of TRIzol (Invitrogen, Carlsbad, CA) using a T 10 basic ULTRA-TURRAX (IKA, Wilmington, NC) and supernatant was kept and separated from lipids by adding chloroform (1vol/5vol) and centrifuging (12000× g, 15 min, 4°C). The aqueous phase was then used to purify RNA using the E.Z.N.A. Total RNA Kit I according manufacturer instructions (Omega Bio-Tek, Norcross, GA). The quantification of RNA was performed using the ND-1000 NanoDrop spectrophotometer (Thermo Fisher Scientific, Rochester, NY) and only those samples displaying a 260/280 absorbance ratio between 1.9 and 2 were utilized. The integrity of RNA was further assessed on a denaturing agarose gel stained with ethidium bromide. Only high-quality RNA, with clear 28S and 18S rRNA bands and with approximately a 2∶1 ratio (28S:18S) was used for later analyses.

### Reverse transcription quantitative polymerase chain reaction (RT-qPCR)

RNA (1 µg) was reverse-transcribed using the M-MLV Reverse Transcriptase (Life Technologies, Burlington, ON) and a C1000 Thermal Cycler (Bio-Rad, Mississauga, ON). All primers for the target and reference genes were designed from guinea pig (cavia porcellus) sequences using NCBI/Primer-BLAST tool (**Table 1**), except GLUT4 (previously published for use in guinea adipose tissue, [47]). The specificity of the primers (three pairs for each gene) was verified by performing melting curve analyses and by subsequent RTq-PCR product size confirmation with a 1.2% agarose gel. Amplification efficiency of the RTq-PCR reaction was determined for each target and for the reference gene using standard curves generated with decreasing concentration of cDNA samples. The amplification efficiencies in all primer sets were 90% to 100%. Quantitative (RTq-PCR) gene expressions were performed with 3 µL of reverse-transcribed RNA using SYBR Green I reagents in a CFX384 Real-Time system instrument (Bio-Rad). Forty cycles of amplification were performed, with each cycle consisting of denaturation at 95°C for 15 s, annealing at 55°C for 15 s and extension at 72°C for 20 s. Control samples containing no cDNA were used to confirm the absence of DNA contamination. Quantification cycle values (Ct) are means of triplicate measurements. In preliminary experiments, β-actin Ct values were observed not to be consistent between the NBW and LBW group (15.3±0.2 versus 16.1±0.2 respectively; p<0.05), whereas glyceraldehyde-3-phosphate dehydrogenase (GAPDH) displayed consistent Ct values between the NBW and LBW groups (21.2±0.3 versus 21.5±0.3 respectively; p = 0.45) and was used as reference gene. The transcript level of target genes was then normalized to the transcript level GAPDH gene. The fold expression of each individual target gene was determined by the 2^−ΔΔCt^ method [48].

**Table 1:** Primers used for analysis of gene expressions by RT-qPCR.

Gene	Accession number*	Forward sequence (5′->3′)	Reverse sequence (3′->5′)
FAS	XM_003464828	CCCAGAAGCAGCTCATTCGC	TGCCCCATGTTGGACTTGGT
ACC1	ENSCPOT00000006107	GTTGGACAACTCCTTCACTC	CAGCCCATCACTTCATCAAA
ACC2	ENSCPOT00000004684	TTATCACGAATGAATCGGGC	AGATCCTTGGTGACATAGGG
ACSL1	NM_001172908	GCAAGCATAGGGAAGGAAAA	TATGGTCTGCAATACGAGGT
DGAT1	XM_003463735	TTTTACAAGCCTCTGGTTCG	GGCTATCATGGCTGTAAAGG
DGAT2	ENSCPOT00000004457	CCTCTCTGTGCTGCAGGTACT	CTGCCACCTTTCTTGGGGGT
ATGL	XM_003461280	CTGCCTGATGTGCCTGAGGA	ACAGCCTGGAGGGTAGGTGA
HSL	XM_003464642	CAGGCCTCGAAGAATGAAAA	GGTGTCTCTGAGTCTAGGTC
FABP4	ENSCPOT00000007262	GCAGATGACAGGAAAGTCAA	GTGACACCATTCATGACACA
CD36	ENSCPOT00000000508	AGGAAAATGTTACAGCGCCT	TCCGGAACATTTGTGCTTTC
HK2	XM_003468966	CTTAGATGACTTCCGCACAG	GCAGTCCACGCTTAGTAAAA
GLUT4	AY949177	GTGGCCATCTTTGGCTTCGTG	CGGCTGAGATCTGGTCAAAC
GLUT1	ENSCPOT00000000640	CAGCAGCAAGAAAGTGACA	GATCCAGGTCTGGTTGTAGA
ADIPOQ	ENSCPOT00000003229	ATGGCACCACGGGCAAATTC	CAGCCTGCTCTCCATTCCCA
MCP-1	NM_001172926	GCTTGTGCTCCAACACTCCA	ACCCACTTCTGTGTGGGGTC
TNFα	U77036	GCCGTCTCCTACCCGGAAAA	TAGATCTGCCCGGAATCGGC
PPARγ1	AF317514	AAGTGCCTTGCTGTGGGGAT	ACTCTGGGTTCAGCTGGTCG
SREBP-1c	ENSCPOT00000002472	ATGGGGGCTACTGTGAAGGC	CTGTGGCCAGGATAGTGCCA
NCoR1	ENSCPOT00000000854	TTAACTACAAAAGGCGGCAC	CTTGACAGCTTCAACTGGTG
GAPDH	EU862201	GCTCGTTTCTTGGTATGACA	CTAGTCTCCATGGTCTCACT

*Accession number for sequences in the National Center for Biotechnology Information (NCBI) database or Ensembl genome browser.

FAS, fatty acid synthase; ACC(1) and (2), acetyl-CoA carboxylase 1 and 2; ACSL1, acyl-CoA synthetase long-chain family member 1; DGAT(1) and (2), diacylglycerol acyltransferase 1 and 2; ATGL; adipose triglyceride lipase, HSL, hormone-sensitive lipase; FABP4, fatty acid binding protein 4; CD36, cluster of Differentiation 36; HK2, hexokinase 2; GLUT4, glucose transporter 4; GLUT1, glucose transporter 1; ADIPOQ, adiponectin; MCP-1, Monocyte chemoattractant protein-1; TNFα, tumor necrosis factor alpha; PPARγ1, peroxisome proliferator-activated receptor-gamma 1; SREBP-1c, sterol-regulatory-element-binding protein-1c; NCoR1, nuclear receptor co-repressor 1; GAPDH, glyceraldehyde 3-phosphate dehydrogenase.

### Quantification of microRNAs (miRs) Expression

Total RNA including the miR fraction was isolated as described above. Reverse transcription of miRs were done according the instruction of the miScript II RT Kit (Qiagen, Toronto, ON). Samples from the NBW and LBW EWAT tissues were run in triplicates using the miScript SYBR Green PCR Kit (Qiagen) in a Bio-Rad CFX384 Real-Time system instrument. Primers for miR expression were all designed and validated by Qiagen and included miR-27a (5′-UUCACAGUGGCUAAGUUCCGC-3′), miR-27b (5′-UUCACAGUGGCUAAGUUCUGC-3′), miR-378 (CUCCUGACUCCAGGUCCUGUGU) miR-24 (5′-UGGCUCAGUUCAGCAGGAACAG-3′), miR-145 (5′-GUCCAGUUUUCCCAGGAAUCCCU-3′), miR-103-2 (5′-AGCAGCAUUGUACAGGGCUAUGA-3′), miR-222 (5′-AGCUACAUCUGGCUACUGGGU-3′), miR-223 (5′-UGUCAGUUUGUCAAAUACCCC-3′) and miR-103-1 (5′-GGCUUCUUUACAGUGCUGCCUUGU-3′). The fold expression of each individual miR target was determined by the 2^−ΔΔCt^ method and normalized to GAPDH expression [49].

### Western Blotting

EWAT samples (∼200 mg each) were homogenized with a T 10 basic ULTRA-TURRAX (Bio-Rad) in 0.8 mL of ice-cold lysis buffer (pH 7.4) containing 10 mmol/L of Tris base (Thermo Fisher Scientific, Whitby, ON), 1 mmol/L of EDTA (Sigma-Aldrich), 0.25 mol/L of sucrose (Thermo Fisher Scientific), protease inhibitor cocktail (EMD Millipore, Billerica, MA) and phosphatase inhibitors (1 M NaF, 0.2 M Na2Vo4). Homogenates were stirred for 1 h on ice using a Tube Rotator (VWR, London, ON) and were then centrifuged at 10,000× g at 4°C for 15 min. The resulting supernatants were collected and total protein concentration of extracts was assessed by D_C_ protein Assay Reagents (Bio-Rad) using BSA (AMRESCO, Solon, Ohio) as a standard reference. Protein extracts were migrated on a Nu-PAGE 4–12% Bis-Tris gel (Life Technologies) and transferred onto Immobilon transfer membranes (EMD Millipore). The membranes were blocked overnight at 4°C with Tris-buffered saline (TBS)/0.1% Tween 20 (Thermo Fisher Scientific) containing 5% skim milk. The blots were then incubated with primary antibodies in TBS/0.1% Tween 20 containing 5% skim milk or albumin bovine serum for 2 h at room temperature. Rabbit antibodies (Santa Cruz Biotechnology, Santa Cruz, CA) against FAS (sc-20140), ACC (C83B10) and the anti-phospho-ACC (Ser79) (#3661), anti-phospho-AMPKα (Thr172) (40H9) antibodies (Cell Signaling Technology, Whitby, ON) were used at a final dilution of 1∶1000. The rabbit anti-phospho-PPARγ (Ser273) (bs-4888R) (Bioss Inc, Woburn, MA) was used at 1/500 final dilution. The blots were thereafter washed in 0.1% Tween 20/TBS and incubated at room temperature for 1 h with secondary rabbit horseradish peroxidase conjugated antibody (1∶10,000) (Cell Signaling Technology) in 0.1% Tween 20/TBS containing 5% skim milk or albumin bovine serum. The mouse β-actin (A 5316) (Sigma-Aldrich) was used at dilution of 1/50,000. Protein bands were revealed by the Luminata Classico Western Blot HRP Substrate (EMD Millipore). The chemiluminescence signal was captured with a Luminescent Image Analyzer (Bio-Rad), and densitometry values (arbitrary units) were determined using the ImageQuant LAS 4000 software (Bio-Rad). The abundance of target proteins was expressed relatively to β-actin protein amount in each sample.

### Plasma Chemistry Analyses

Fasted glucose, total cholesterol and triglyceride levels in the plasma were quantitated by colorimetric assays using reagents (kits No. 11447513216, 11491458216 and 11877771216 respectively) from Roche Diagnostics (Laval, QC). All the assays were run on Cobas Mira S autoanalyzer (Laval, QC). The intra-assay CV for glucose, total cholesterol and triglycerides measurements were 1.7, 1.9, and 2% respectively. The inter-assay CV for glucose, total cholesterol and triglycerides measurements were 1.5, 3.2 and 2.6% respectively.

### Statistical analysis

Data are presented as means ± SEM. Statistical significance was determined using a two-tailed unpaired *t*-test with GraphPad Prism 5 (GraphPad Software, San Diego, CA). Values of p≤0.05 were considered statistically significant.

## Results

### Growth performance, energy intake and plasma metabolites

**Figure 1:**
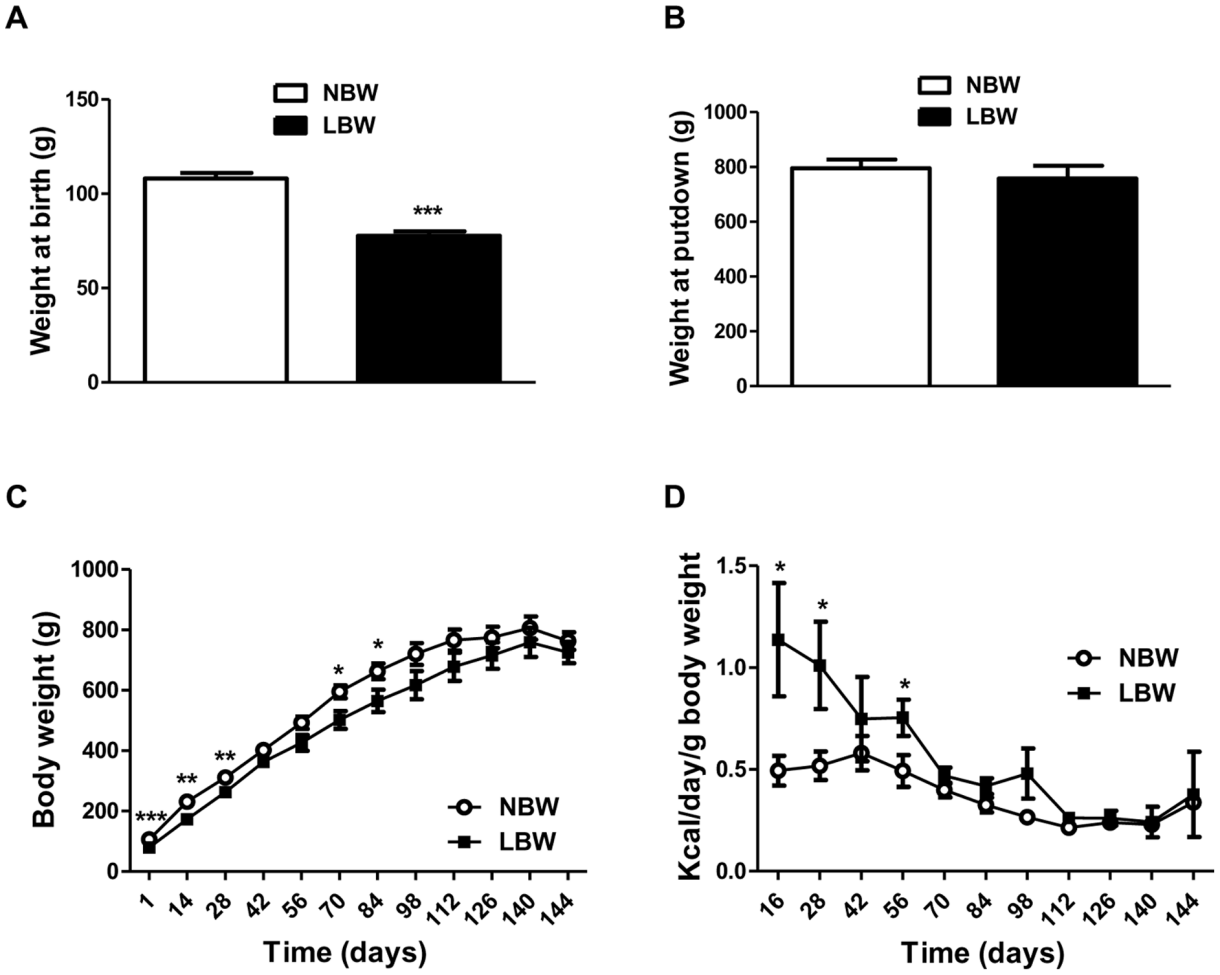
Growth performance and food intake of animals throughout the experiment. **A and B**, the weights of animals at birth and put down respectively. **C**, the weights of the animals throughout the experiment. The open circles represent NBW animals; and the closed squares, LBW animals. **D**, Food intake of animals from weaning to putdown. Data are the means ± SEM (n = 7 for NBW and n = 5 for LBW). Significance was determined by a two-tailed unpaired Student's *t*-test. *p<0.05, **p<0.01, ***p<0.001.

The body weight of LBW pups at birth was 28% less than that of NBW pups (p<0.001). LBW pups continued to display a low weight compared to NBW throughout early postnatal life (day 1 to day 98) (**Figures 1A and 1C**). From day 98 to the end of the experiment (day 145) corresponding to early adulthood, body weights were similar between NBW and LBW animals (**Figure 1B and 1C**). During the early post weaning period up to day 60, the relative daily energy intake (kcal/day/g body weight) was higher in LBW compared to NBW guinea pigs (p≤0.05), but did not differ significantly (p>0.10, **Figure 1D**) thereafter. Plasma concentrations of glucose and triglycerides measured at 145 days of age were not significantly different in NBW (10.43±2.71 and 1.38±0.40 mmol/L respectively) versus LBW (18.45±4.26 and 1.24±0.27 mmol/L respectively, p>0.10). Plasma total cholesterol was increased by 32% in LBW (1.08±0.09 mmol/L) compared to NBW (1.42±0.12 mmol/L), although this was not statistically significant (p = 0.063).

### EWAT relative weight, lipid content and cell size were increased in LBW offspring while, whole body composition was unchanged

**Figure 2:**
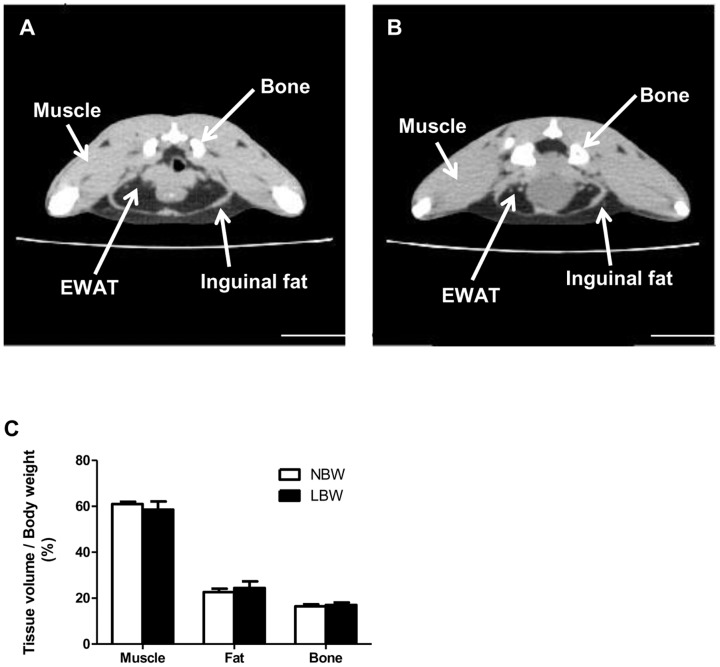
CT analysis demonstrated no changes in total bone, muscle and adipose tissue in LBW offspring. Guinea pigs were anesthetized and scanned from the proximal tibia to the base of the skull and images were taken at 1.25**A**, a CT-scan image slice of a NBW; **B**, a CT-scan image slice of a LBW. Adipose tissue is characterized by a lower intensity signal (darker regions on the image). The area occupied by the bone, epididymal (EWAT) and inguinal fats are indicated. **C**, Volumes occupied by muscle, fat and bone in the area between the proximal tibia to the base of the skull was calculated using an application developed in MATLAB, normalized to body weight and expressed as a percentage of total body volume. The values represent means ± SEM; n = 5 for NBW and n = 4 for LBW. Significance was determined by a two-tailed unpaired Student's *t*-test.

**Figure 3:**
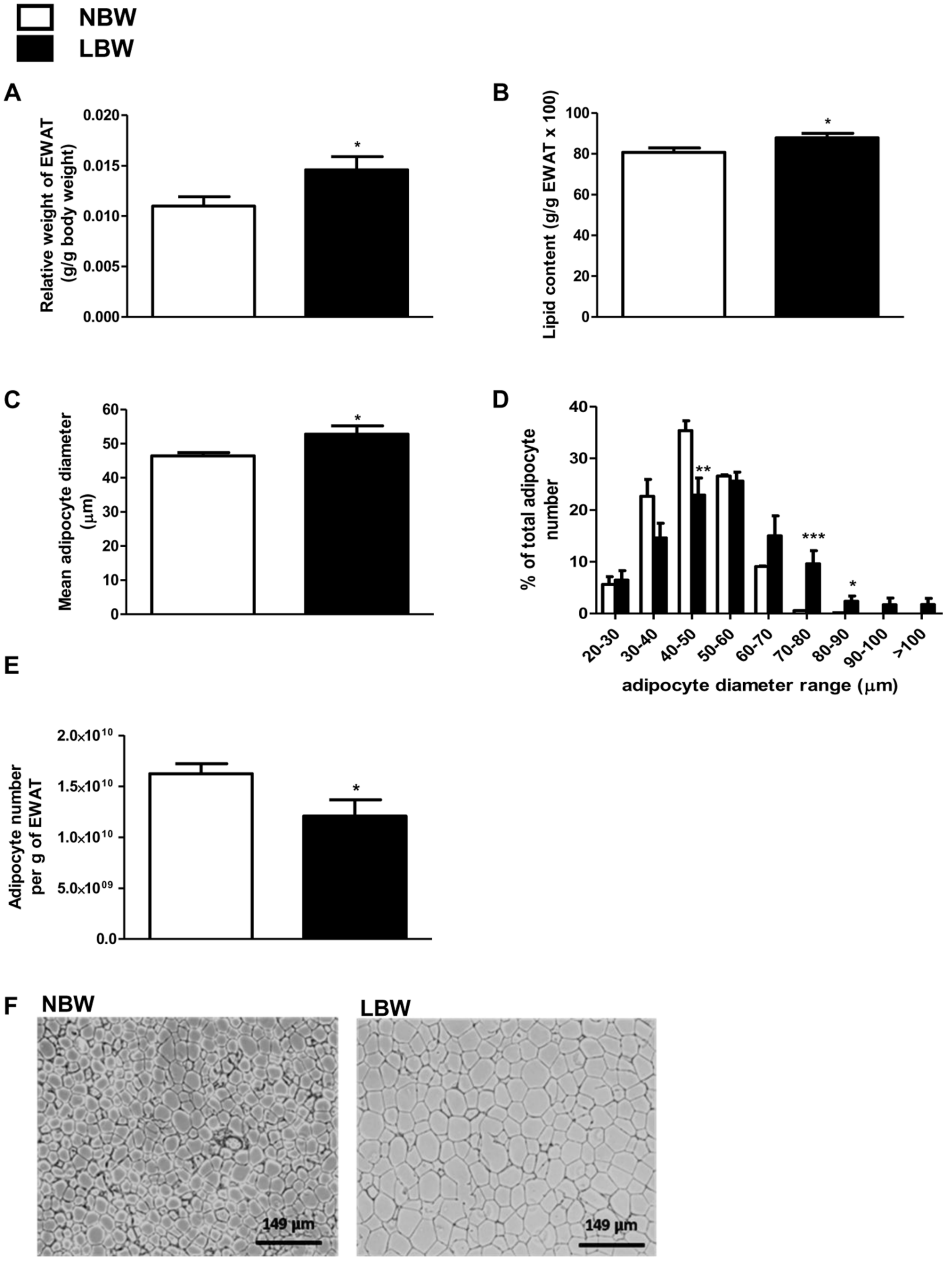
EWAT relative weight, lipid content and cell size were increased in LBW at 145 days. **A**, white epididymal adipose tissue (EWAT) adjusted by body weight; **B**, epididymal fat cellular lipid content; **C**, mean epididymal adipocyte diameter; **D**, epididymal adipocyte distribution based on cell diameter; **E**, epididymal cell number per gram of EWAT; **F**, representative pictures of EWAT (magnification 10×). Data are the means ± SEM (n = 7 for NBW and n = 5 for LBW). Significance was determined by a two-tailed unpaired Student's *t*-test. *p<0.05, **p<0.01, ***p<0.001.

To assess the body composition in NBW and LBW offspring, CT scans were performed at ∼120 days of age (**Figures 2A and 2B**). The total relative volume of muscle, fat and bone for the area between the proximal tibia to the base of the skull was similar between LBW and NBW offspring (**Figure 2C**). Interestingly however, at tissue collection (145 days), the relative weight of the EWAT (g/g body weight) was 36% greater in LBW compared to NBW (p<0.05, **Figure 3A**). The amount of lipid [(g per g of EWAT)×100] was slightly greater (+9%, p = 0.05) (**Figure 3B**) and the mean epididymal adipocyte diameter was increased by 15% (p<0.05) in LBW compared to NBW (**Figures 3C and 3F**). The percentage of epididymal small adipocytes with a diameter between 40 and 50 µm was lower (p<0.01) and the percent of large adipocytes with a diameter between 70 and 90 µm was greater (p<0.05), in LBW relative to the NBW group (**Figure 3D**). Consequently, the number of cells per gram of EWAT was significantly lower (−26%, p<0.05) in LBW compared to NBW animals (**Figure 3E**).

### Genes involved in lipid synthesis and storage were increased in EWAT of LBW

**Figure 4:**
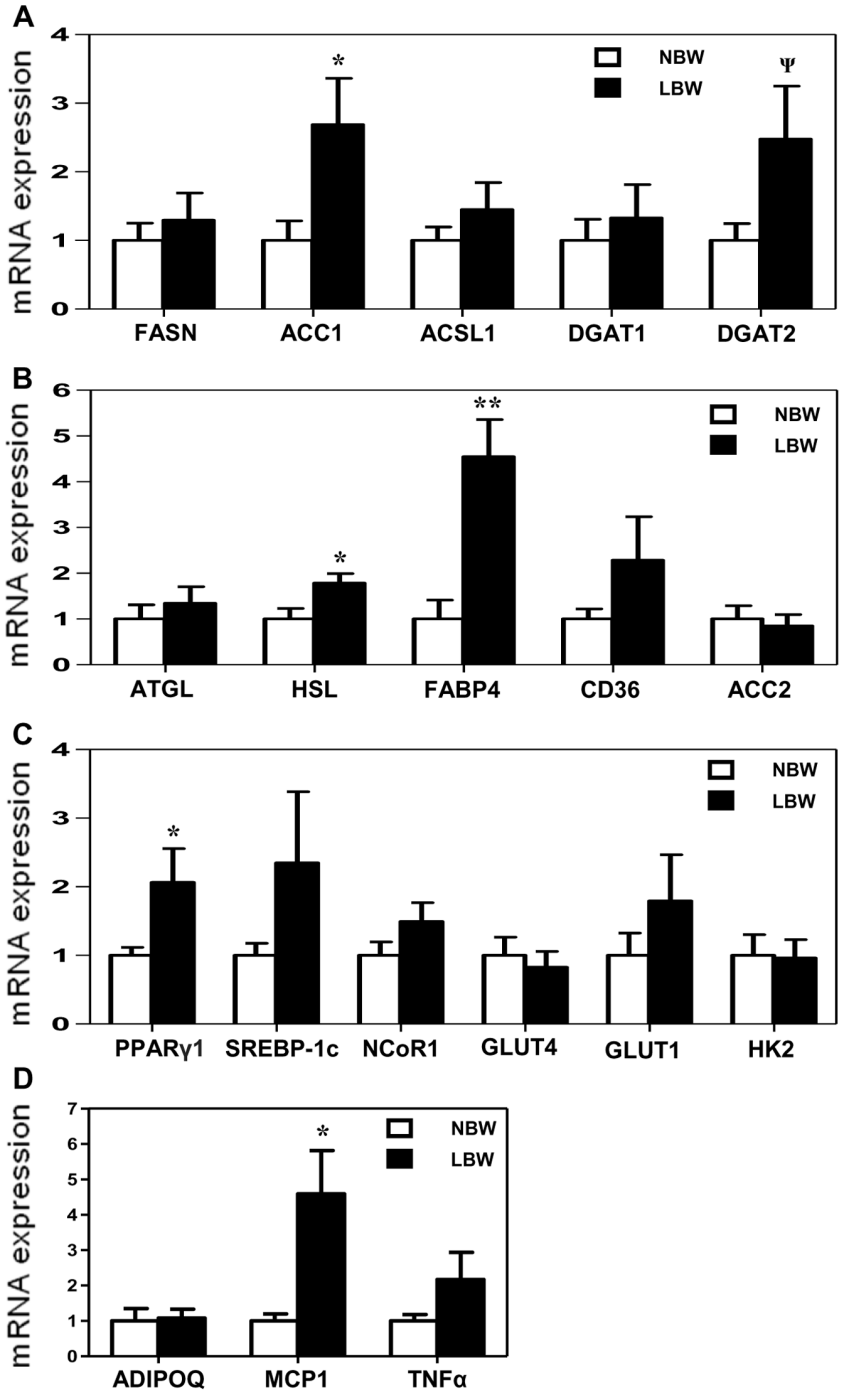
Expression of genes involved in lipid synthesis and storage was induced in EWAT of LBW. mRNA expressions of lipogenic genes (**A**), genes involved in fatty acids mobilization and transport (**B**), nuclear transcriptional regulators and genes of glucose metabolism (**C**) and adipokines (**D**). The fold expression of each individual target gene was calculated against GAPDH mRNA transcript. mRNA expressions in LBW are presented relatively to mRNA expressions of NBW. Data are the means ± SEM; n = 7 to 6 for NBW and n = 5 to 4 for LBW. Significance was determined by a two-tailed unpaired Student's *t*-test. Ψp<0.10, *p<0.05, **p<0.01.

ACC1 is involved in adipose tissue development and lipid synthesis. The mRNA expression of ACC1 was higher in LBW EWAT compared to NBW offspring (p<0.05, **Figure 4A**). Additionally, the mRNA expression of DGAT2, a catalyzer of triglyceride synthesis was increased in EWAT of LBW, although this did not reach statistical significance (p = 0.09, **Figure 4A**). Furthermore, the mRNA expression of HSL, a key enzyme in the regulation of lipid stores, and FABP4, a factor in lipid trafficking in adipose tissue, was significantly elevated in EWAT of LBW relative to NBW group (p<0.05 and 0.01 respectively, **Figure 4B**). Additionally, the mRNA expression of PPARγ1, a regulator of adipogenesis as well as lipid synthesis in adipose tissue, was significantly higher in LBW EWAT (p<0.05, **Figure 4C**). However the mRNA expression of other nuclear transcriptional regulators (SREBP-1c and NCoR1) also involved in lipid metabolism regulation, was unaltered. The mRNA expression of downstream PPARγ/SREBP-1c lipogenic targets, FASN, ACSL1 and DGAT1, was unchanged between NBW and LBW (**Figure 4A**). Furthermore mRNA expression of ATGL (involved in triglyceride breakdown), CD36 and ACC2 (implicated in fatty acid uptake and oxidation), as well as GLUT4, GLUT1 and HK2 (components of glucose metabolism) in EWAT did not differ between LBW and NBW offspring (**Figure 4B, 4C**). Finally, a significant increase in mRNA expression of the Monocyte chemotactic protein-1 (MCP1) was also observed in LBW EWAT (p<0.05) although, mRNA expression of the adipokines (ADIPOQ and TNFα) was unaltered (**Figure 4D**).

### EWAT phospho-ACC (Ser79), phospho-AMPKα (Thr172) and phospho-PPARγ (Ser273) were decreased in LBW

**Figure 5:**
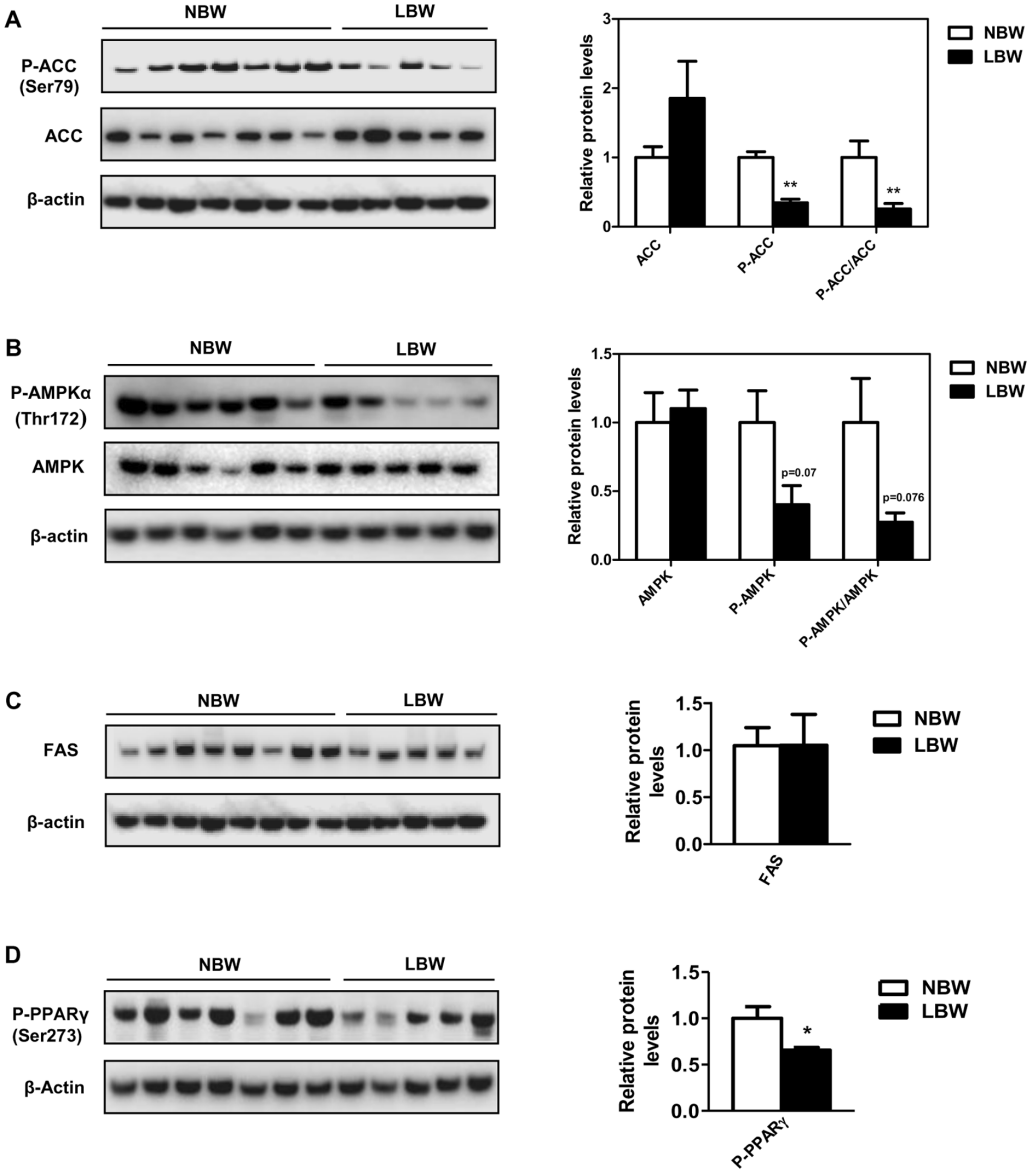
EWAT phospho-ACC (Ser79), phospho-AMPKα (Thr172) and phospho-PPARγ (Ser273) were decreased in LBW. The left panel shows representive Western blots and the right panel shows summarized data of densitometric analyses. **A**, abundance of phospho-ACC (Ser79) and total ACC proteins. **B**, abundance of phospho-AMPKα (Thr172) and total AMPK proteins. **C** and **D**, abundance of FAS and phospho-PPARγ (Ser273) proteins. The protein levels were normalized to that of β-actin protein levels. Data are the means ± SEM (n = 7 to 6 for NBW and n = 5 for LBW). Significance was determined by a two-tailed unpaired Student's *t*-test. *p<0.05, ***p<0.001.

Post-translational modification of proteins, including phosphorylation contributes to the regulation of lipid metabolism. To further characterize the molecular and metabolic changes associated with the increased EWAT in LBW, we investigated the phosphorylation status of the key regulators in fatty acid biosynthesis, AMPK, ACC and PPARγ. In LBW offspring EWAT, there was a 3.9-fold decrease (p<0.001) of the phospho-ACC (Ser79), while total ACC protein was not significantly different (p = 0.102, **Figure 5A**) between the 2 groups. Consistent with the decreased phospho-ACC, phospho-AMPKα (Thr172) tended to be lower in the EWAT of LBW versus NBW (−73%, p = 0.076; **Figure 5B**). Total AMPK protein was comparable and not significantly different between the two groups (**Figure 5B**). In addition, LBW animals had a significant 1.5-fold reduction in phospho-PPARγ (Ser273), (p = 0.047, **Figure 5D**). Levels of FAS protein were not different between experimental groups (p = 0.989, **Figure 5C**).

### The expression of miR-24 and miR-103-2 was increased in EWAT of LBW

**Figure 6:**
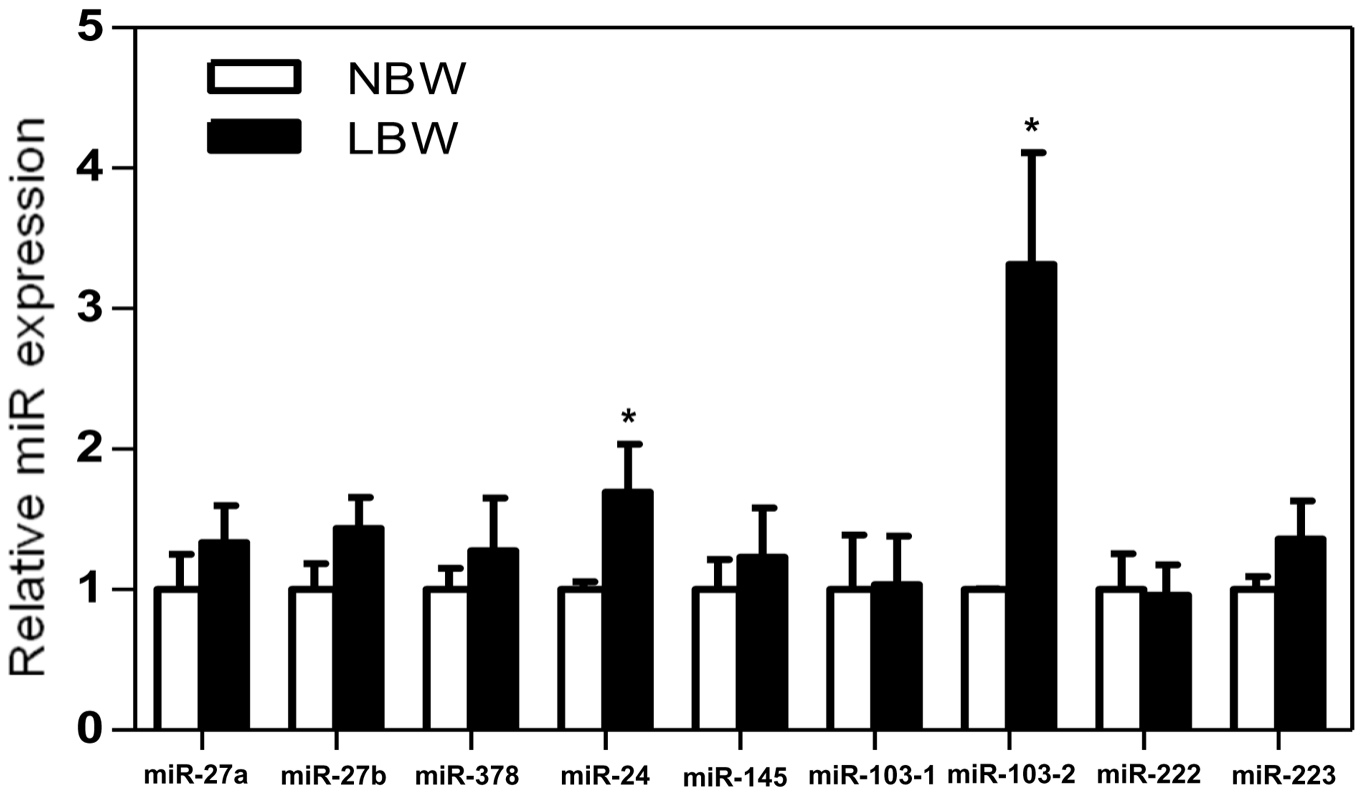
miR-24 and miR-103-2 were induced in EWAT LBW at 145 days of age. miR-27a, miR-27b, miR-378, miR-24, miR-145, miR-103-1, miR-103-2, miR-222 and miR-223 all related to adipose tissue development were measured by RT-qPCR. The fold expression of each individual miR target was calculated against GAPDH mRNA transcript. miR expressions in LBW (n = 5) are presented relatively to miR expression of NBW (n = 7). Significance was determined by a two-tailed unpaired Student's *t*-test. *p<0.05.

In order to assess potential additional mediators of the increased relative EWAT weight associated with LBW, the expression of nine miRs related to adipose tissue development was examined (**Figure 6**). Interestingly, LBW was associated with increased expression of miR-24 (+70%, p<0.05) and miR-103-2 (+231%, p<0.01) related to adipogenesis and adipocyte size, respectively. The expression of other miRs related to adipogenesis (miR-27a, miR-27b, miR-145, and miR-222), lipogenesis (miR-378), cell size (miR-103-1) and macrophage activation (miR-223) in adipose tissue was not significantly different in EWAT of LBW versus NBW at this age (**Figure 6**).

## Discussion

The present study demonstrates that LBW, induced by uterine artery ablation, is associated with postnatal increased lipid deposition and cellular hypertrophy in EWAT, a major visceral depot in the guinea pig. Importantly, by early adulthood, there was an absence of changes in whole body composition and, in particular a lack of increased overall body fat. It also reveals persistent changes in the expression of genes and miRs and post-translational modifications, known to regulate lipid accumulation in postnatal life. Together, these results support the concept of an *in utero* programmed dysregulation of lipid synthesis and accumulation in visceral adipose tissue of young adult LBW male offspring. They also suggest that these observed molecular changes are contributors rather than consequences of the increased visceral obesity reported in human LBW offspring. The most striking novelty of the present study is that, following an adverse *in utero* environment, LBW offspring display increased lipid synthesis and adipocyte hypertrophy in visceral adipose tissue, in conjunction with an altered lipogenic protein phosphorylation status and miR expression pattern.

Increased relative weight of EWAT was found in 145-day-old LBW male guinea pig offspring, independent of changes in whole body composition. A similar observation has also been reported in a rat UPI-induced IUGR model where the epididymal fat pad mass at the age of 30 weeks was significantly increased, yet body weight was unaltered [50]. In addition to the increased EWAT mass in LBW male offspring reported in the current study, the mean diameter of epididymal adipocytes was also increased, indicating cell enlargement within this tissue, presumably associated with increased lipid storage. Indeed, LBW offspring displayed higher lipid content in the epididymal adipose tissue compared to NBW. The current observation of hypertrophic adipocytes is consistent with adult-onset of adipocyte enlargement demonstrated in the 50% food-restricted IUGR male rat model [39], suggesting that dysregulation of adipocyte morphology in epididymal adipose tissue can be programmed *in utero*. In sum, the above observations underscore the potential involvement of prenatal growth in regulation of fat distribution and morphology.

Several possible mediators of the observed hypertrophic adipocytes have been examined in the current study. We provide evidence that the increase in lipid content and adipocyte hypertrophy in EWAT is attributed to the increased mRNA expression of ACC1 in conjunction with decreased phospho-ACC (Ser79). In adipose tissue, increased phosphorylation of ACC at Ser79, inhibits ACC activity and thereby reduces production of malonyl-CoA, the substrate of fatty acid synthesis [10], [51], [52]. It has also been reported that activation of adipose AMPKα via specifically phosphorylation at Thr172, results in phosphorylation of ACC at Ser79, which inhibits fatty acid synthesis [10]. Therefore, the decrease in phospho-AMPKα (Thr172) and subsequent decrease in phospho-ACC (Ser79) in conjunction with the up-regulation of ACC1 mRNA may be associated with the promotion of fatty acid synthesis and cell enlargement in EWAT of LBW. Although not significant, the increased mRNA expression of lipogenic DGAT2 gene found in EWAT of LBW offspring, could also contribute to the increased lipid formation and thereby the adipocyte hypertrophy in this fat deposit in early adulthood. Further protein analysis would be needed to confirm this hypothesis.

While our data implicate lipogenic genes as being involved in the increased EWAT in young adult LBW guinea pig, we expanded our studies to investigate the possibility that genes involved in utilization of non-lipid substrates such as glucose, for *de novo* synthesis of fatty acids in adipocytes, might also contribute. Despite the disproportionate increase in EWAT mass and evidence of increased lipid content in EWAT in LBW offspring, there were no changes in the mRNA expression of the glucose transporters GLUT4 and GLUT1, nor in the glycolytic gene HK2, in LBW offspring EWAT. The lack of changes in this suite of genes occurred in conjunction with unchanged fasting circulating glucose levels. Based on this, we speculate that changes in basal glucose uptake into EWAT are unlikely to have made a substantial contribution to increase lipid synthesis and subsequent EWAT deposition in LBW offspring. This is supported by previous studies, which have shown that unlike rat fat cells, guinea pig epididymal fat cells utilize acetate and lactate as lipogenic substrates and poorly utilize glucose [53], [54]. However, while glucose may not play a major role in guinea pig fat, we cannot rule out the possibility that acetate and lactate incorporation into epididymal adipocytes is not altered in LBW offspring.

In adipose tissue, lipolysis is regulated by a series of lipases, with ATGL being the main lipase at the initial step of triglyceride hydrolysis and HSL acting at a sequential step to hydrolyze diglycerides [26], [55]. In the present study, the mRNA expression of HSL, but not ATGL, was increased in the EWAT of LBW compared to NBW offspring. This increase in HSL expression is in agreement with the increased visceral adipose HSL expression that has been reported to occur in nutritional-deficiency IUGR rat offspring, [39]. In this previous study, an increased release of fatty acids from IUGR adipocytes likely contributing to elevated plasma fatty acids levels has been reported as a consequence of the increased adipose HSL. Interestingly, this does not appear to be the case in the guinea pig, as plasma triglyceride levels were unchanged between LBW and NBW offspring. As 60% of fatty acids resulting from adipose tissue lipolysis are re-esterified in fasted rats [56] and re-esterification of fatty acids from intraadipocyte lipolysis may participate in the adipocyte triglyceride synthesis [22], it would appear that the increase in HSL expression in EWAT of LBW contributes to the increased lipid content observed in this tissue. In addition, the increased mRNA expression of FABP4 in EWAT of LBW guinea pigs also supports the idea that increased adipocyte HSL expression contributes to the increased generation of free fatty acids for lipid synthesis and storage in epididymal adipocytes of LBW offspring. Indeed, FABP4 affects intracellular lipid metabolism by transporting fatty acids or fatty acid substrates to the nucleus for transcriptional regulation, to mitochondria for β-oxidation, and to lipid droplets for storage as triglycerides [23].

Having shown the involvement of AMPK/ACC axis, DGAT2, FABP4 and HSL in the increased lipid content observed in EWAT of LBW offspring, the roles of additional molecular signaling mechanisms were explored. The ligand-activated nuclear transcriptional factor PPARγ is expressed in adult adipocytes and regulates adipogenesis and genes involved in all pathways of lipid metabolism, including fatty acid synthesis, resulting in increased fat deposition [13], [57], [58]. Phosphorylation status is key in regulating protein activity and especially as it relates to adipogenesis and lipogenesis [10], [59], [60]. Adipocyte-specific Nuclear Receptor Corepressor (NCoR) knockout (AKO) mice exhibit a decrease in cyclin-dependent kinase (Cdk5)-mediated PPARγ (Ser273) phosphorylation in both epidididymal adipose tissue and primary adipocytes, resulting in constitutive activation of an increase of PPARγ-responsive genes. Such a decrease in phospho-PPARγ was accompanied by an increased mRNA expression of PPARγ and hyperplasia within the visceral epidididymal adipose tissue [14]. Interestingly, in LBW EWAT, there was reduced phospho-PPARγ (Ser273) and increased mRNA expression of PPARγ1, despite any changes in the mRNA expression of PPARγ cofactor SREBP-1c and PPARγ corepressor NCoR1. Further, adipocyte hypertrophy, but not hyperplasia was observed. Thus a programmed increased PPARγ1 mRNA and decreased phospho-PPARγ (Ser273), induced in situations of limited nutrient supply in utero, may be involved in the increased expression of ACC1 and therefore, activation of lipid synthesis within EWAT. Indeed, the adipogenic/lipogenic transcription factor PPARγ regulates the expression of a number of downstream lipogenic genes in epididymal adipose tissue including ACC [13]. Further, given that a non-genomic role for PPARγ is emerging [61], the decrease in phospho-PPARγ (Ser273) as an *in utero* programmed effect, is likely an important factor in the EWAT phenotype of LBW offspring. Such effects appear not to involve NCoR1 or SREBP-1c gene expression although further investigations are needed to shed light on the specific factor that promotes decreased phosphorylation of PPARγ in later life LBW EWAT.

In addition to post-translational modification, miRs have been reported to play a role in lipogenesis, adipocyte size and adipogenesis. Over-expression of miR-103 in goat mammary gland epithelial cells leads to increased transcription of genes associated with goat mammary gland fat synthesis, including PPARγ, ACC1 and FAS [62]. Conversely, silencing of miR-103 decreases total fat by reducing adipocyte size in mice [17], suggesting that it plays a role in regulating adipocyte size. Another miR involved in adipose tissue accumulation, miR-24, is increased in epididymal adipocytes from leptin deficient ob/ob and diet-induced obese (DIO) [15]. Other studies have reported miR-24 involvement in adipogenesis [16], [18]. In the current report, we observed increased miR-24 and miR-103-2 in EWAT of young adult LBW offspring. The mechanisms underlying this programmed elevation later in life following an adverse *in utero* environment are yet to be determined. However, several hypoxia-regulated miRs including miR-24, miR-103-1 and miR-103-2 have been studied in carcinogenesis and have been shown to promote cell survival in hypoxic microenvironments [63]. Therefore, it is possible that the increase in miR-103-2 and miR-24 expression is an *in utero* programmed mechanism in a hypoxic situation, such as occurs with uterine artery ablation [64], which could increase adipocyte fatty acid synthesis through activation of PPARγ and ACC1 within this tissue. Future studies should seek to clarify the exact role of miR-24 and miR-103-2 in visceral adipocyte hypertrophy in LBW offspring and to identify their direct associations with PPARγ or other genes involved in lipid metabolism such as ACC1, DGAT2, FABP4 and HSL.

In summary, these data indicate that LBW induced by UPI is associated with an increase in visceral adipose tissue deposition that involves an active modulation of genes, miRs and post-translational modifications, which impact lipid anabolism and storage in male guinea pigs in early adulthood. Our findings support the concept of *in utero* programming of postnatal visceral adipose tissue properties, independent of whole body compositional changes and shed light on the potential underlying mechanisms resulting in postnatal visceral adipose tissue accumulation following development in an adverse *in utero* environment.
